# Deep venous thrombosis (DVT) diagnostics: gleaning insights from point-of-care ultrasound (PoCUS) techniques in emergencies: a systematic review and meta-analysis

**DOI:** 10.1186/s13089-024-00378-1

**Published:** 2024-07-30

**Authors:** Hany A. Zaki, Bilal Albaroudi, Eman E. Shaban, Mohamed Elgassim, Nood Dhafi Almarri, Kaleem Basharat, Ahmed Shaban

**Affiliations:** 1 Department of Emergency Medicine, Hamad Medical Corporation, Doha, Qatar; 2 Department of Cardiology, Al Jufairi Diagnosis and Treatment, MOH, Doha, Qatar; 3 Department of Internal Medicine, Mansoura General Hospital, Mansoura, Egypt

**Keywords:** Deep vein thrombosis, Deep venous thrombosis, Emergency medicine, Meta-analysis, Point-of-care systems, Ultrasound, Sensitivity, Specificity, Systematic review

## Abstract

**Background:**

The assessment of deep venous thrombosis (DVT) is clinically difficult diagnosis. The “gold standard test” for DVT diagnosis is venography; however, various point-of-care ultrasound (POCUS) protocols have been suggested for DVT evaluation in the emergency department.

**Aims:**

This review evaluated the role of different POCUS protocols in diagnosing DVT in the emergency department.

**Methods:**

A systematic review and meta-analysis was conducted based of PRISMA guideline and registered on PROSEPRO (CRD42023398871). An electronic database search in Embase, PubMed, ScienceDirect, and Google scholar and a manual search were performed to identify eligible studies till February 2023. Quality Assessment of Diagnostic Accuracy Studies tool (QUADAS-2) was used to assess the risk of bias in included studies. Quantitative analysis was carried out using STATA 16 and Review Manager software (RevMan 5.4.1). Sensitivity, specificity of POCUS protocols for DVT diagnosis compared to reference standard test was calculated.

**Results:**

Heterogeneity was identified between 26 included studies for review. The pooled sensitivity, specificity, PPV, and NPV for the 2-point POCUS protocol were 92.32% (95% CI: 87.58–97.06), 96.86% (95% CI: 95.09–98.64), 88.41% (95% CI: 82.24–94.58) and 97.25% (95% CI: 95.51–98.99), respectively. Similarly, the pooled sensitivity, specificity, PPV, and NPV for 3-point POCUS were 89.15% (95% CI: 83.24–95.07), 92.71% (95% CI: 89.59–95.83), 81.27% (95% CI: 73.79–88.75), and 95.47% (95% CI: 92.93–98). The data pooled for complete compression ultrasound, and whole-leg duplex ultrasound also resulted in a sensitivity and specificity of 100% (95% CI: 98.21–100) and 97.05% (95% CI: 92.25–100), respectively. On the other hand, the time from triage to DVT diagnosis was significantly shorter for emergency physician-performed POCUS than diagnostic tests performed by radiologists.

**Conclusion:**

The diagnostic performance of POCUS protocols performed by emergency physicians was excellent. Combined with the significant reduction in time to diagnosis. POCUS can be used as the first-line imaging tool for DVT diagnosis in the emergency department. We also recommended that attending emergency physicians with POCUS training are present during DVT diagnosis to improve diagnostic performance even though high diagnostic performance is observed even with the minimum training.

**Supplementary Information:**

The online version contains supplementary material available at 10.1186/s13089-024-00378-1.

## Introduction

Deep venous thrombosis (DVT) is an obstructive disease that hinders the mechanism of venous reflux. It is one of the common venous thromboembolic (VTE) disorders, with an incidence rate of 1.6 per 1000 yearly [1]. The cause of DVT is usually associated with the following risk factors; reduced blood flow as a result of immobility (bed rest, general anesthesia, operations, strokes, and long flights) [2, 3], increased venous pressure due to mechanical compression or functional impairments [4], mechanical injury to the vein such as trauma, surgery, peripherally inserted catheters and intravenous drug abuse [5] and increased blood viscosity due to polycythemia rubra vera, thrombocytosis and dehydration [6]. The diagnosis of DVT in the emergency department (ED) should be fast and accurate to avoid the clinical progression to pulmonary embolism, the most feared complication leading to a high mortality rate of these patients [7].

The clinical diagnosis of DVT is difficult; thus, imaging is usually required. Venography is considered the gold standard for DVT diagnosis; however, over the past 20 years, multiple point-of-care ultrasound (POCUS) protocols for DVT evaluation have been developed. The most common protocols are the 2-point and 3-point compression techniques. The 2-point technique, which is commonly used, tests the compressibility of the common femoral vein (CFV) and the popliteal vein (PV), while the 3-point technique involves testing the CFV, superficial femoral vein (SFV), and PV compressibility. Other protocols used in DVT diagnosis include the complete proximal leg compression technique, which involves the compression of every 1–2 cm along the entire visible length of CFV and PV, and the whole-leg compression technique, which involves compressing the calf veins alongside the CFV and PV. However, these protocols consume more time than the 2 and 3-point protocols [8].

The main aim of this systematic review and meta-analysis was to assess the diagnostic performance of POCUS protocols used to diagnose DVT and carried out in the emergency department (ED) or by emergency physicians (EP).

## Methodology

### Protocol and registration

This systematic review and meta-analysis was conducted in accordance with the Preferred Reporting Items for Systematic Reviews and Meta-Analyses (PRISMA) 2020 guiding principles and protocol registered on PROSPERO article (CRD42023398871)**.**

### Data sources and search strategy

The Embase, PubMed, ScienceDirect, and Google scholar databases were scoured for scientific articles published between January 1, 2000, and February 2023. The search involved combining keywords such as Deep vein thrombosis and point-of-care ultrasound with the Boolean expressions “AND” and “OR” to form a detailed search strategy. Furthermore, additional studies were identified by snowballing and hand searching of key medical journals. Full details about the search strategy employed in each electronic database is outlined in Appendix A.

### Study selection

The search was restricted to studies on humans and published in English. For studies to be eligible for inclusion, two reviewers had to ensure that they fulfilled the following criteria.

1. Studies designed as either observational or randomized trials.
2. Studies evaluating different protocols of POCUS in the diagnosis of DVT.
3. Studies in which the diagnosis was carried out by an emergency physician or in the emergency department.
4. Studies reporting at least one of the following results; specificity, sensitivity, negative predictive value (NPV), positive predictive values (PPV), or time to DVT diagnosis.

Studies were excluded for the following reasons;

1. Studies that only assessed the POCUS protocols for DVT diagnosis in the radiology department or by a radiologist.
2. Studies designed as either systematic reviews and meta-analyses, case reports, letters to the editor, or guidelines.
3. Studies with less than 50 participants. This criterion was critical in ensuring that the statistical power of our meta-analysis was upheld.

### Data extraction

Two reviewers tasked with data extraction compiled all the relevant data from the included studies in (Table 1). The data compiled included Author ID (first author’s surname and the year of publishing), study design, location of the trial or study, participants’ characteristics including the sample size, gender distribution, and mean age, the ultrasound machine used, the reference standard for DVT diagnosis and main outcomes. The main outcomes retrieved for use in the current study were specificity, sensitivity, NPV, and PPV values, while the secondary outcomes were time from triage to DVT diagnosis. Discrepancies in the extracted data were reconciled through a discussion between the two reviewers or by consulting a third reviewer.

**Table 1:** Study characteristics

Author ID	Study design	Location	Participants’ characteristics	US Machine specifications	Reference standard	POCUS protocol	Main outcomes
Garcia et al. [9]	Prospective cross-sectional study	Spain	109 patients (49 male and 60 females; mean age: 68 + 16 years	Esaote MyLab 25 with a 7.5MHz linear probe	Radiologist DUS	3-point	POCUS evaluation had a sensitivity, specificity, positive LR, and negative LR of 93.2%, 90%, 9.32, and 0.08, respectively
Abbasi et al. [10]	Prospective cross-sectional study	Iran	81 patients (46 males and 35 females; mean age: 47.2 ± 18.6 years.)	Honda HS-2000	Radiologist DUS	3-point	The overall POCUS evaluation had a sensitivity, specificity, positive LR, and negative LR of 85.9%, 41.2%, 1.5, and 0.3, respectively
Jang et al. [11]	Prospective study	United States	72 patients (48 female and 24 males; mean age: 54 years)	Aloka SSD-1400 with a 7.5MHz linear array probe	Radiologist DUS or contrast venography	3-point	The sensitivity and specificity of EMR performed POCUS were 100% and 91.8%, respectively
Kim et al. [12]	Prospective diagnostic study	United States	296 patients (147 male and 149 females; median age: 50 (37–60) years)	Toshiba SSH-140A with a 7.5MHz linear transducer	Radiologist DUS	3-point	The POCUS evaluation had a sensitivity, specificity, positive LR, and negative LR of 86%, 97%, 26.5, and 0.14, respectively
Shiver et al. [13]	Prospective study	United States	61 patients (41 females and 20 males; mean age: 43 years)	Phillips HD1 4000 or SonoSite MicroMaxx with a broadband linear array 12.5MHz transducer	Contrast venography	3-point	The overall sensitivity and specificity for diagnosing DVT were 86% and 100%, respectively
Kline et al. [14]	Prospective single-center study	United States	185 patients (109 female and 76 males; mean age: 51.6 + 16.1 years)	Ultrasonix CEP or Ultrasonix Corp with a 14.5MHz linear-format broadband probes	Radiologist US	3-point	The EP-performed US showed a high positive LR of 6.5 The EP-performed US showed a sensitivity and specificity of 70% and 89%, respectively
Fischer et al. [15]	Prospective cohort study	United States	73 patients (49 males and 24 females; median age 61 (26–92) years)	Sonosite M-turbo, Sonosite Nanomaxx and Sonosite S-Fast	Radiology FSV	3-point	The sensitivity, specificity, and positive and negative LR of the POCUS test were 100%, 95.8%, 23.4, and 0, respectively
Seyedhosseini et al. [16]	RCT	Iran	50 patients (29 male and 21 female)	Sonoace X8, Medison with a high-frequency US probe	Radiologist US	3-point	The specificity and sensitivity of POCUS were 100%
Crowhurst et al. [17]	Prospective study	Australia	178 patients (90 male and 88 females; median age: 57 (21–96) years)	Sonosite Micromaxx with 7.5–10MHz linear array transducer	Radiologist DUS	3-point	The sensitivity and specificity of POCUS were 77.8% and 91.4% respectively
Dehbozorgi et al. [18]	Prospective cross-sectional study	Iran	240 patients (120 male and 120 females; mean age: 59.46 + 16.58 years)	Fujifilm Sonosite with 6–15 MHz linear probe	Radiologist DUS	3-point	The POCUS test had a sensitivity and specificity of 100% and 93. 33% respectively
Jahanian et al. [19]	Prospective cross-sectional study	Iran	72 patients (36 male and 36 females; median age: 36 + 19 years)	Sonoace Medison	Radiologist DUS	3-point	The diagnostic performance of POCUS evaluation was 53.8% sensitivity and 85.7% specificity
El-Gazzar et al. [20]	Prospective cross-sectional study	Egypt	100 patients	Portable DC-30 with a 7.5–10 MHz linear array transducer and 3.5MHz for obese patients	Radiologist DUS	3-point	The POCUS exam had a sensitivity and specificity of 94.12% and 92.42%, respectively
Zuker-Herman et al. [21]	Prospective study	Israel	195 patients (77 males and 118 females; mean age: 66.09 ± 16.79 years)	Phillips US device with a 7.5 MHz linear array probe	Radiologist DUS	3-point and 2-point	2POCUS examination diagnosed DVT with a sensitivity, specificity, positive LR, and negative LR of 82.76%, 98.52%, 55.865, and 0.18%, respectively 3POCUS examination diagnosed DVT with a sensitivity, specificity, positive LR, and negative LR of 90.57%, 98.52%, 96.00%, and 96.38%, respectively
Crisp et al. [22]	Prospective cross-sectional study	United States	188 patients	Bard-Site-Rite IV with a 7.5MHz linear probe	Radiologist DUS	2-point	The sensitivity and specificity of the EP-performed US was 100% and 99%, respectively
Reihani et al. [23]	Cross-sectional study	Iran	63 patients (30 female and 33 males; mean age: 54.71 (22–96) years)	NR	Radiologist DUS	2-point	The overall POCUS sensitivity and specificity for DVT diagnosis was 86.6% and 80.4%
Farahmand et al. [24]	Prospective study	Iran	74 patients (41 male and 33 females; mean age: 55.16 + 17.4 years)	FF Sonic UF-4300R FUKUDA DENSHI with a 7.5 MHz linear array transducer	Radiologist DUS	2-point	The POCUS was 100% specific and 100% sensitive
Frazee et al. [25]	Prospective observational study	United States	76 patients (48 male and 28 females; mean age: 49 years)	Aloka 650 CL with 7.5MHz probe or 3.5MHz probe for obese patients	Radiologist DUS	2-point	The ED POCUS had a sensitivity and specificity of 88.9% and 75.9%, respectively
Jacoby et al. [26]	Prospective study	United States	121 patients	ATL 5000 with a 7.5 MHz vascular probe	Radiologist DUS	2-point	POCUS carried out by Emergency residents had a sensitivity and specificity of 89% and 97%, respectively
Canakci et al. [27]	Retrospective study	Turkey	266 patients (142 females and 124 males; median age: 63 (48–74) years)	GE logic E/ Vivid E 2013	Radiologist US or Venography	2-point	POCUS examination had a sensitivity, specificity, positive and negative LR of 93%, 93%, 14, and 0.08, respectively
Poley et al. [28]	Cross-sectional observational study	Canada	184 patients (137 females and 47 males; mean age: 56 + 18 years)	Esaote MyLab 5 with 10–12.5 MHz linear array probe	Radiologist DUS	2-point	The specificity and sensitivity of POCUS were 91% and 97%, respectively
Pujol et al. [29]	Prospective study	United States	56 patients (33 women and 23 men; mean age: 73 (59–84) years	V-Scan Dual probe, GE	Radiologist DUS	2-point	The US exam diagnosed DVT with a specificity and sensitivity of 100%
Theodoro et al. [30]	Prospective study	United States	156 patients	Agilent Image point Hx with 10 MHz linear probe or Sonosite 180 with 10.5 MHz linear array probe	Radiologist DUS	2-point	A high correlation with radiology results was observed (0.9 kappa and 99% agreement)
Torres-Macho et al. [31]	Prospective study	Spain	76 patients with suspected DVT	Siemens Sonoline G-20 with a 5–10 MHz linear probe	Radiologist DUS	2-point	The bedside ultrasound diagnosed DVT with 92% sensitivity and 98% specificity
Zitek et al. [32]	Prospective study	United States	234 patients (119 males and 115 females; median age: 48 (18–85) years)	Mindray M7 with a 7.5MHz linear probe	Radiologist US	2-point	The sonographic findings of EP for DVT diagnosis resulted in 57.1% sensitivity and 96.1% specificity
Magazzini et al. [33]	Prospective study	Italy	399 patients (212 female and 187 females; mean age: 64.5 + 18 years)	Esaote US device with 10MHz linear array transducer	Radiologist DUS	CCUS	DVT was diagnosed with 100% sensitivity and 98.5% specificity
Blaivas et al. [34]	Prospective study	United States	112 patients	Aloka 2000 with 5.0 MHz linear array probe	Radiologist DUS	Whole-leg DUS	The recorded sensitivity and specificity was 100% and 98.7%, respectively

*POCUS* Point-of-care ultrasound, *US* ultrasound, *DUS* Duplex ultrasound, *CCUS* complete compression ultrasound, *LR* likelihood ratio, *DVT* Deep venous thrombosis, *ED* Emergency Department, *EP* Emergency physicians

### Quality assessment

The risk of bias was assessed using the Quality Assessment of Diagnostic Accuracy Studies (QUADAS-2) tool provided in the Review Manager software (RevMan 5.4.1). This framework consists of 2 categories (Assessment of bias and applicability concerns). The risk of bias category is further subdivided into four domains which include patient selection, index test, reference standard, and flow and timing, while the applicability concerns is subdivided into patient selection, index test, and reference standard.

### Data synthesis

STATA 16 software was used in the calculation of the overall specificity, sensitivity, PPV, and NPV values, while the RevMan software was utilized in the analysis of the overall effect of POCUS in time from triage to DVT diagnosis. The DerSimonian-Laird random effect model was implemented when pooling both the primary and secondary outcomes since it has the ability to take into account the expected heterogeneity. The summarized estimates of POCUS were then plotted forest plots for each outcome. Heterogeneity was also calculated using the I^2^ statistics, of which the values were categorized as follows; 0–40%, low heterogeneity; 41–60%, moderate heterogeneity; and 61–100, substantial heterogeneity. Further analysis was done to check for the significance, of which a p-value of less than 5% (p < 0.05) was considered significant statistically. Additionally, a meta-regression analysis was carried out to identify sources of heterogeneity. In the regression analysis, we classified the level of training into experienced and inexperienced. Inexperienced was used to refer to emergency physicians (EP) who received POCUS training for less than three months, while experienced referred to EP who received POCUS training for more than three months or those who had carried out a sufficient number of POCUS examinations before the trial (at least 50 previous POCUS exams).

## Results

### Study selection

**Fig. 1:**
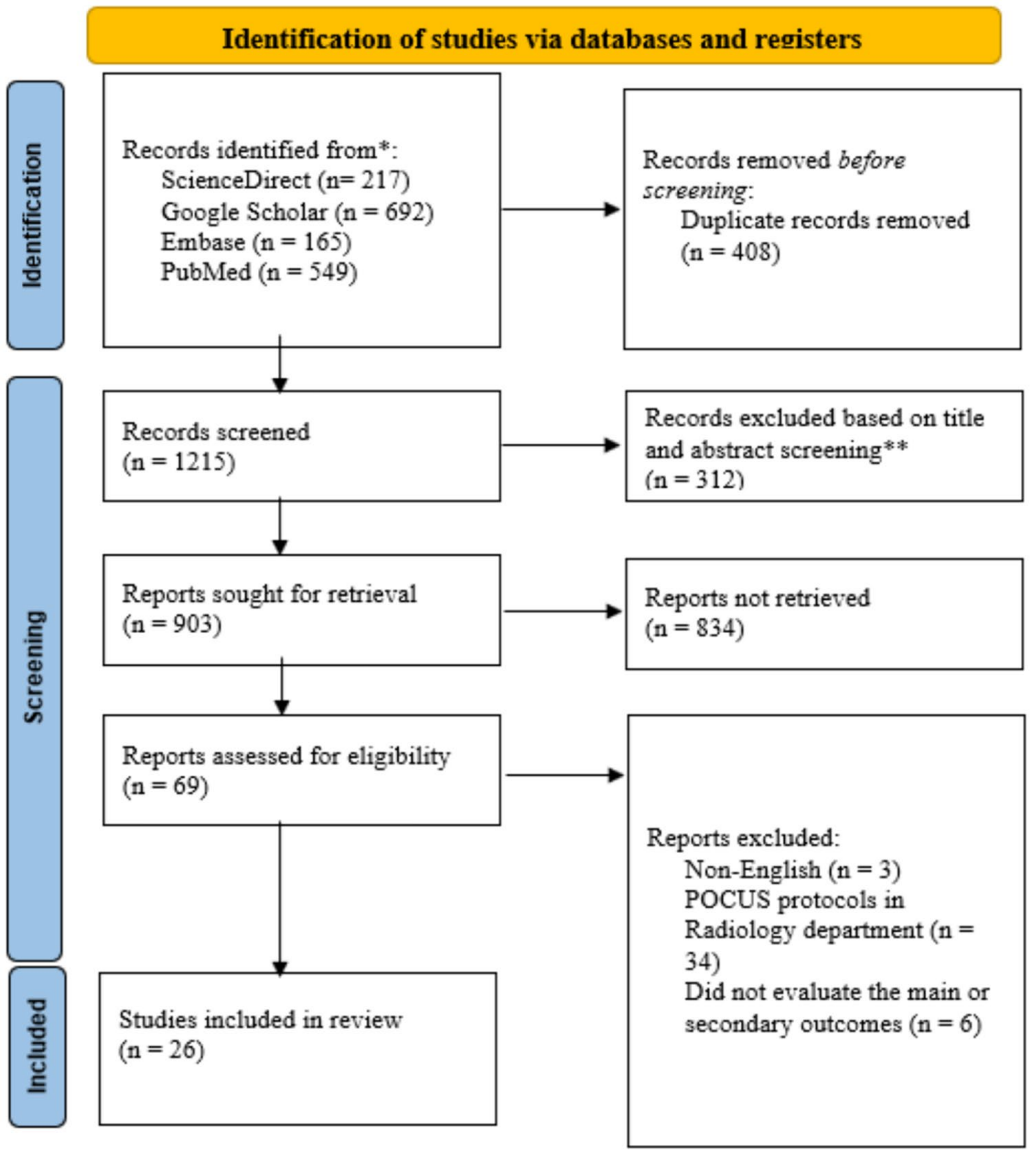
PRISMA flow diagram for study selection

A total of 1623 articles were identified and screened. These articles first underwent a duplicate check, of which 408 were deemed close or exact duplicates and excluded. The remaining 1215 articles were screened by going through the titles and abstracts, of which 312 were excluded. Out of the 903 articles remaining, we did not retrieve 834 because they were either article published before 2000, abstracts without full articles, diagnostic algorithm studies, case reports, and systematic reviews. Finally, we included only 26 studies [9–34] while the other studies were excluded as follows; 3 were observational studies published in different languages, 34 were studies carried out in the radiology department, and 6 did not evaluate either one of the main or secondary outcomes of this review. The complete literature selection is presented in the PRISMA diagram below (Fig. 1).

### Quality assessment results

**Fig. 2:**
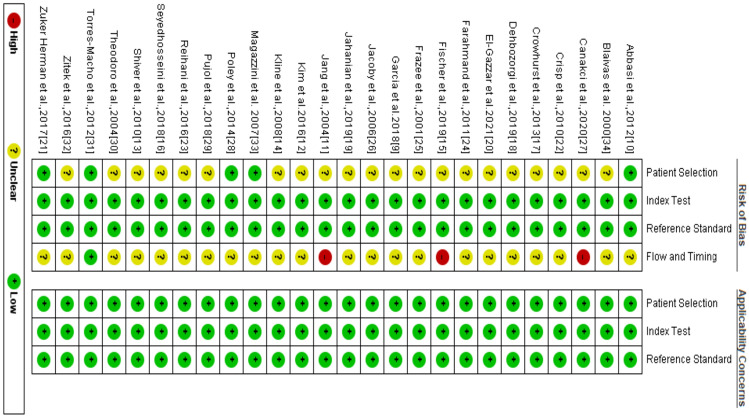
QUADAS-2 bias assessment summary

**Fig. 3:**
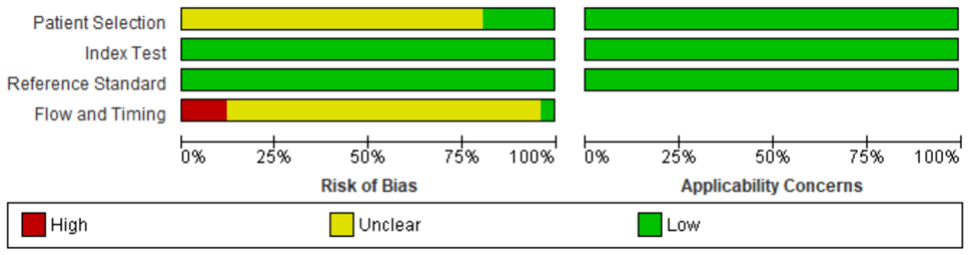
Risk of bias and applicability concerns graph

The risk of bias assessment results are summarized in Figs. 2 and 3 below. Overall, the QUADAS-2 tool revealed that all studies had a low risk of bias and low concern since the studies satisfied at least four of the 7 evaluation criteria. In regard with patient selection, we noticed most of the studies had an unclear risk bias since they employed the convenience sampling method rather than consecutive sampling. However, a low concern was associated with the patient selection. Similarly, most of the studies had an unclear risk of bias about the flow and timing because they did not specify the interval between POCUS and reference tests. Three studies showed a high risk of bias about flow and timing since they evaluated more than one reference tests. All the domains in the applicability section showed a low concern.

### Diagnostic performance of 3-point POCUS for DVT

**Fig. 4:**
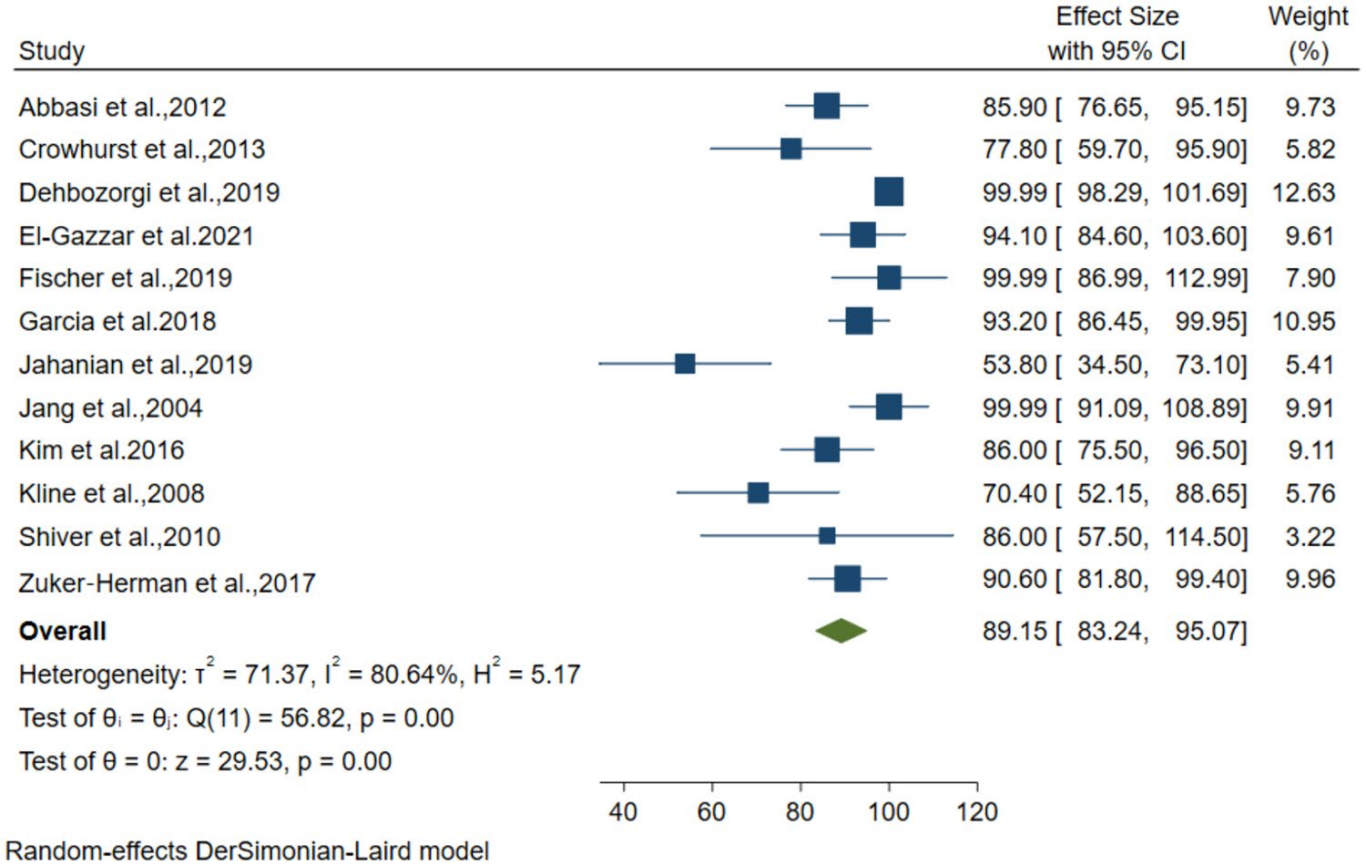
Forest plot of pooled Sensitivity of 3-point POCUS in diagnosing DVT

**Fig. 5:**
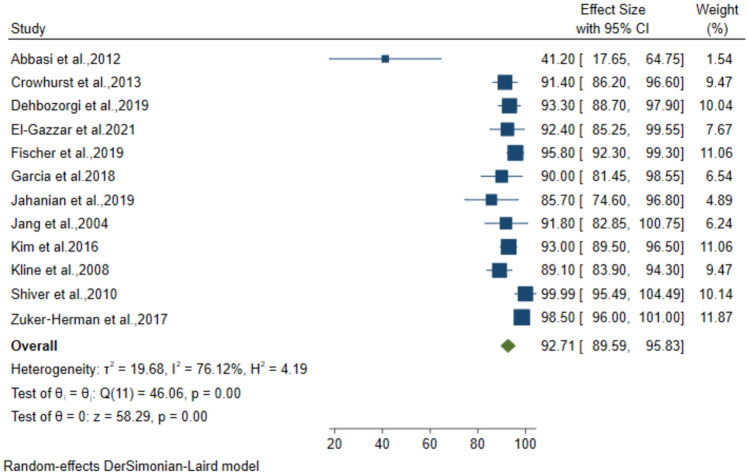
Forest plot of pooled Specificity of 3-point POCUS in diagnosing DVT

**Fig. 6:**
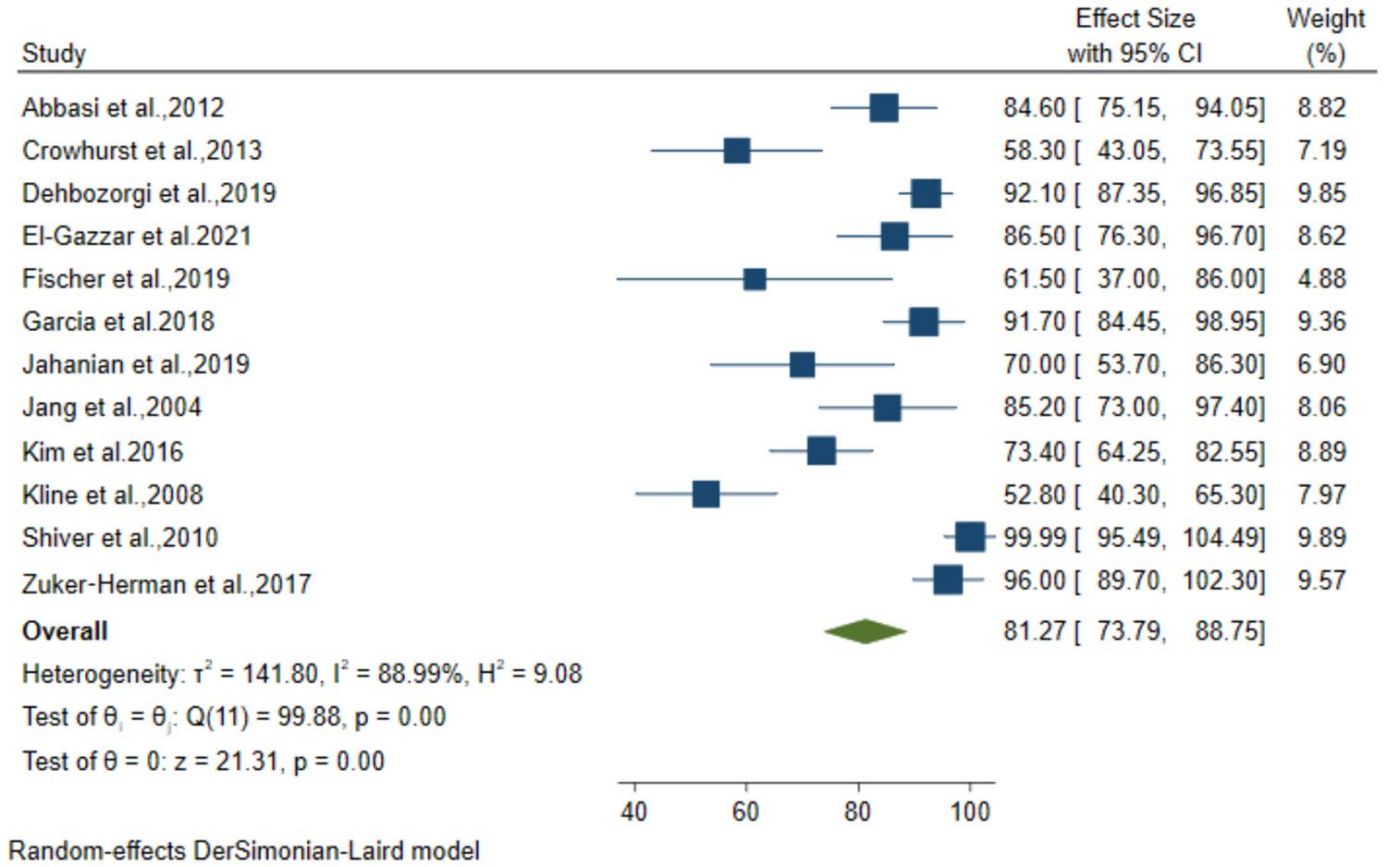
Forest plot of pooled PPV of 3-point POCUS in diagnosing DVT

**Fig. 7:**
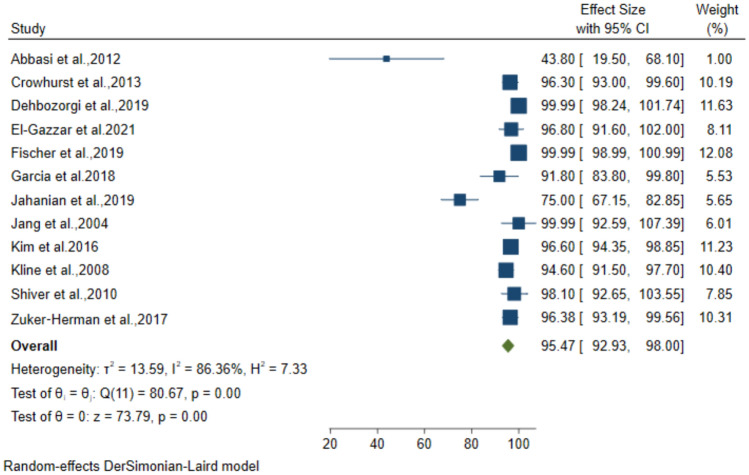
Forest plot of pooled NPV of 3-point POCUS in diagnosing DVT

Twelve studies including 1662 patients with suspected DVT, used the 3-point compression protocol for diagnosis. The pooled data from these studies resulted in 89.15% (95% CI: 83.24–95.07) sensitivity, 92.71% (95% CI: 89.59–95.83) specificity, 81.27% (95% CI: 73.79–88.75) PPV and 95.47% (95% CI: 92.93–98) NPV for the diagnosis of DVT (Figs. 4, 5, 6, 7).

### Diagnostic performance of 2-point POCUS for DVT

**Fig. 8:**
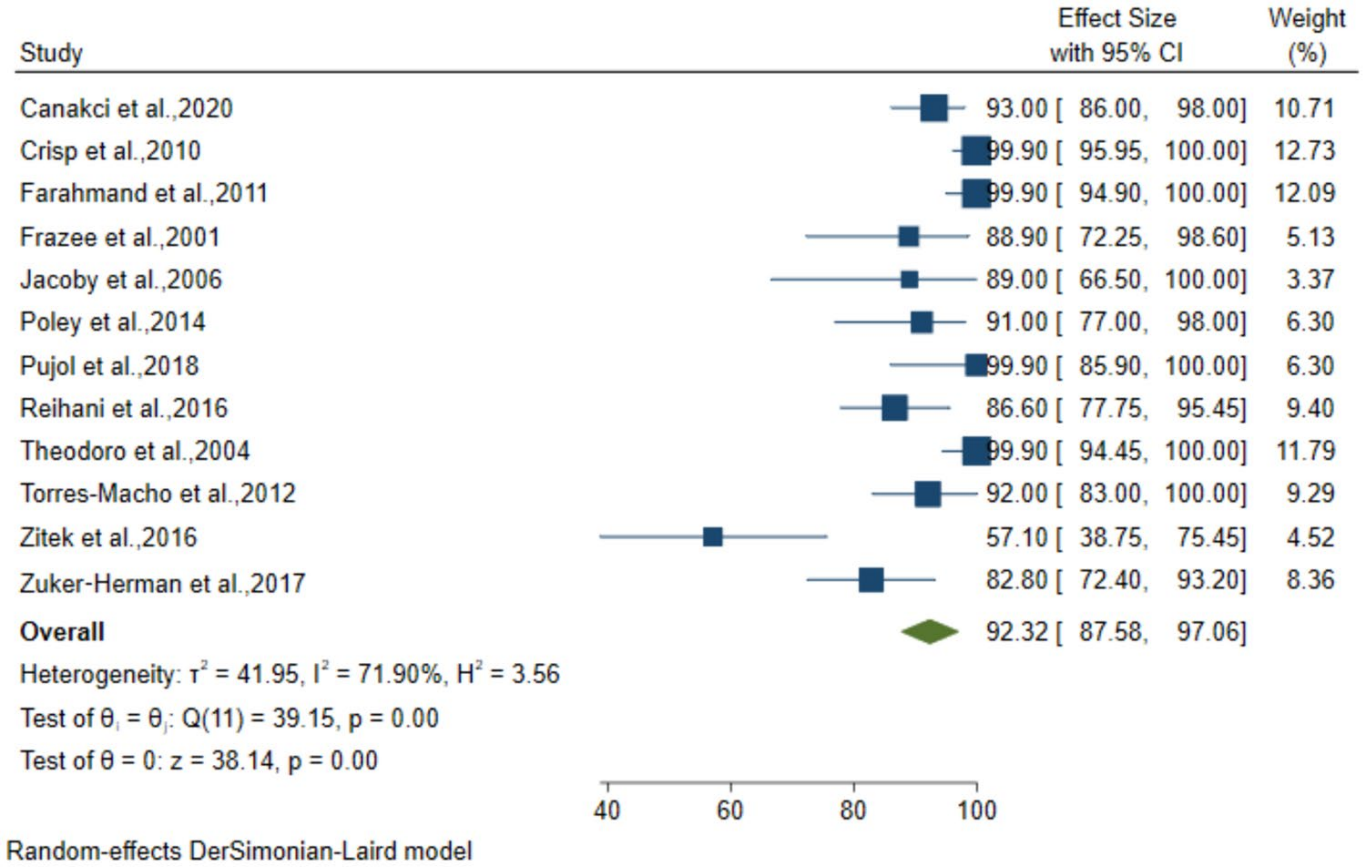
Forest plot of pooled Sensitivity of 2-point POCUS in diagnosing DVT

**Fig. 9:**
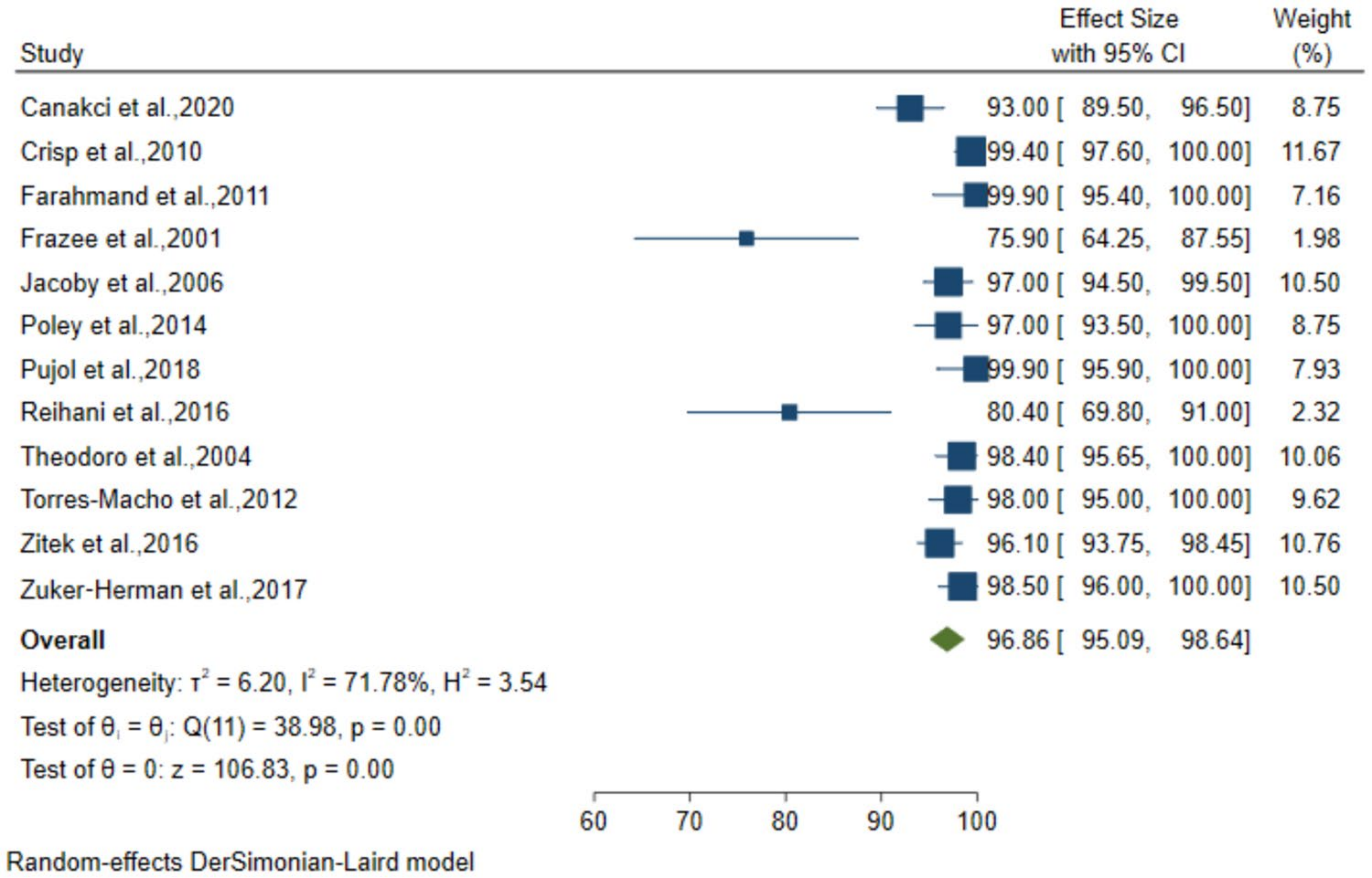
Forest plot of pooled Specificity of 2-point POCUS in diagnosing DVT

**Fig. 10:**
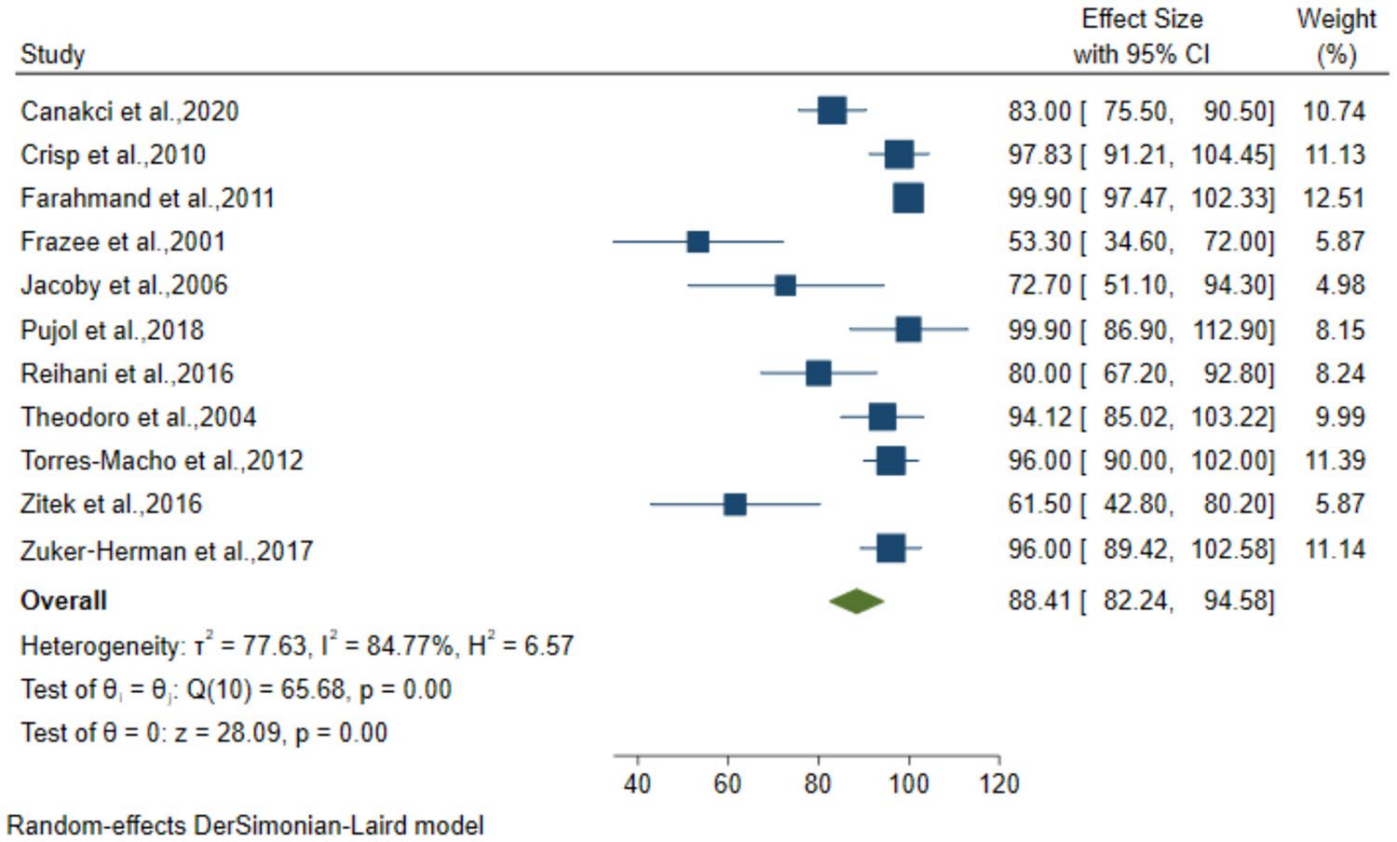
Forest plot of pooled PPV of 2-point POCUS in diagnosing DVT

**Fig. 11:**
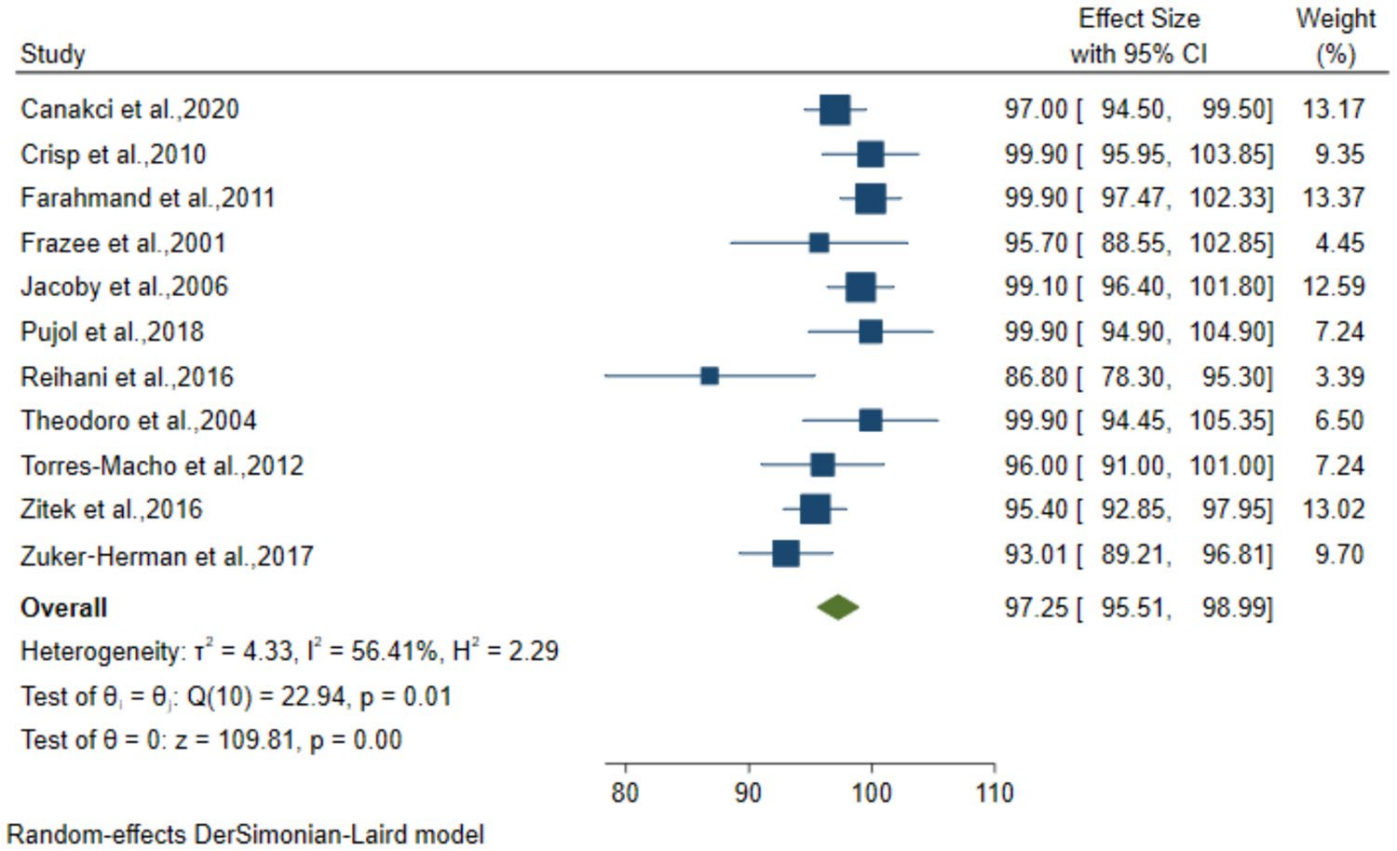
Forest plot of pooled NPV of 2-point POCUS in diagnosing DVT

The 2-point Compression technique was employed in 12 studies which included 1689 patients suspected to have DVT. The pooled sensitivity, specificity, PPV, and NPV for the diagnosis was 92.32% (95% CI: 87.58–97.06), 96.86% (95% CI: 95.09–98.64), 88.41% (95% CI: 82.24–94.58) and 97.25% (95% CI: 95.51–98.99), respectively (Figs. 8, 9, 10, 11).

### Diagnostic performance of Other POCUS protocols

Only two studies in this review evaluated other POCUS protocols (Complete compression ultrasound and whole-leg duplex ultrasound) for DVT diagnosis in the ED. The pooled data from these studies yielded a sensitivity and specificity of 100% (95% CI: 98.21–100) and 97.05% (95% CI: 92.25–100), respectively.

### Time from triage to diagnosis

**Fig. 12:**
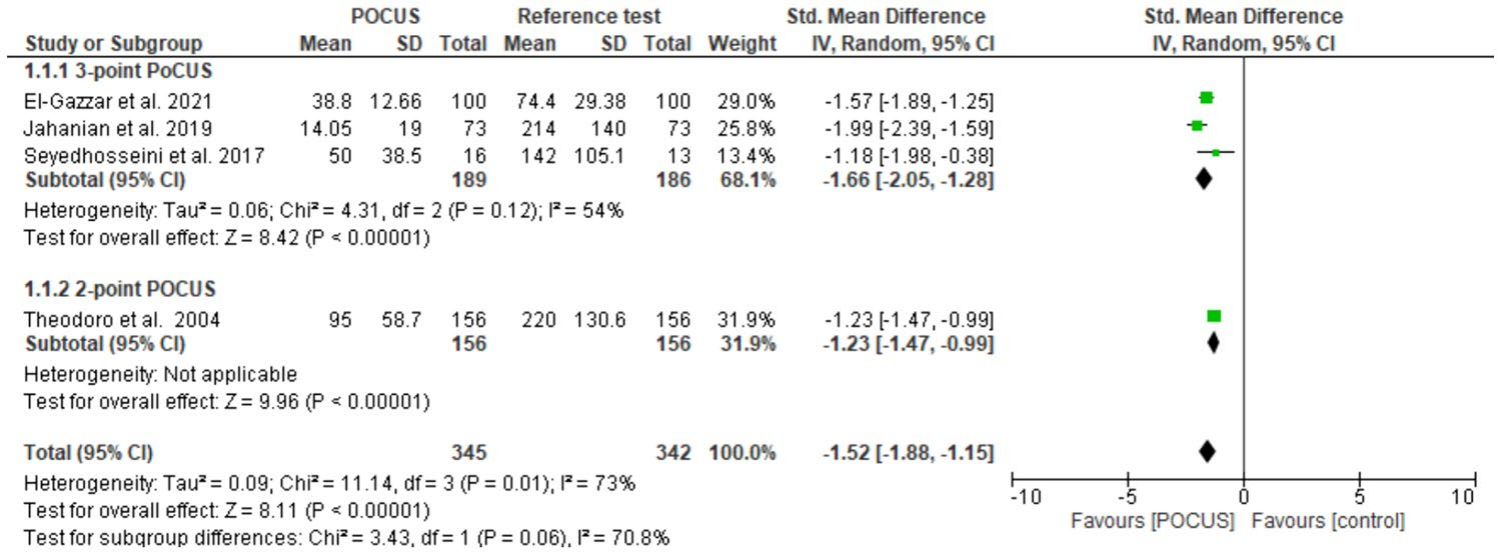
A forest plot showing time from triage to DVT diagnosis

Three studies employing the 3-point protocol and one utilizing the 2-point protocol reported the time taken to make a diagnosis from triage. The pooled data shows that the time taken from triage to diagnosis was significantly shorter when the emergency physicians carried out the 3-point and 2-point POCUS compared to the reference tests carried out by radiologists (SMD: -1.52; 95% CI: -1.88, 1.15) (Fig. 12). All data related to time was represented in minutes.

### Meta-regression analysis

The meta-analysis results for 2 and 3-point POCUS have shown high heterogeneity; therefore, a regression analysis was conducted to identify the sources of heterogeneity. The analysis showed that the heterogeneity in the specificity of the 3-point POCUS was contributed by the type of reference test used (p = 0.0237), while the significant source of heterogeneity in sensitivity was the initial POCUS performer (p = 0.0194). In the 2-Point POCUS protocol, the only identified considerable source of heterogeneity was the number of emergency physicians conducting the tests. The other covariates, including the level of POCUS training, the continent from which the study was conducted, and the sampling method, did not show any significant impact on either POCUS protocol (Table 2).

**Table 2:** Meta-regression analysis of the potential sources of heterogeneity

Covariates	No.of Studies	Sensitivity	Significance (p-value)	Specificity	Significance (p-value)
3-Point POCUS					
Level of POCUS Training					
Experienced	3	93.23 (87.83–98.64)	0.5344	93.23 (87.83–98.64)	0.4378
Unexperienced	9	87.41 (79.55–95.26)		91.88 (88.15–95.61)	
Reference Test					
Contrast venography	2	98.75 (90.25–100)	0.3331	96.85 (89.04–100)	0.0237
Duplex	8	89.67 (82.63–96.70)		91.72 (87.33–96.10)	
Radiologist Ultrasound	2	80.06 (65.21–94.91)		91.54 (87.85–95.24)	
Continent for the Study					
North America	5	90.28 (79.91–100)	0.8925	94.31 (90.79–97.82)	0.9148
Asia	4	85.45 (72.21–98.70)		87.11 (77.14–97.08)	
Europe	1	93.2 (83.8–97.3)		90 (78.6–95.7)	
Africa	1	94.1 (80.3–99.3)		92.4 (83.2–97.5)	
Australia	1	77.8 (54.8–91)		91.4 (84.9–95.3)	
Number EPs					
≥ 10	4	84.63 (75.17–94.08)	0.3236	91.56 (89.13–93.99)	0.9256
< 10	6	89 (79.55–98.44)		91.85 (85.65–98.05)	
Sampling					
Consecutive	2	88.37 (81.99–94.74)	0.2247	74.10 (45–100)	0.5431
Convenience	7	94.66 (89.19–100)		94.28 (91.88–96.68)	
Initial POCUS performer					
Independent EM residents	3	78.19 (50.43–100)	0.0194	90.68 (86.52–94.85)	0.0691
EM residents supervised by attending EP	6	89.72 (81.96–97.48)		91.30 (86.03–96.57)	
2-point POCUS					
Level of POCUS Training					
Experienced	5	94.46 (88.73–100)	0.5296	95.77 (92.33–99.22)	0.4974
Inexperienced	7	90.04 (82.58–97.50)		97.45 (95.32–99.58)	
Reference Test					
Duplex	8	93.66 (88.37–98.95)	0.5274	96.83 (94.23–99.42)	0.7495
Radiologist Ultrasound	4	88.73 (77.72–99.73)		96.52 (94.38–98.66)	
Continent for the Study					
North America	6	90.79 (82.11–99.47)	0.8506	96.88 (94.54–99.21)	0.9931
Asia	3	90.43 (79.02–100)		95.15 (88.34–100)	
Europe	3	93.60 (88.46–98.74)		96. 92 (93.04–100)	
Number EP					
≥ 10	2	74.59 (41.39–100)	0.0004	96.38 (94.43–98.33)	0.9953
< 10	9	93.63 (89.21–98.05)		96.21 (93.66–98.75)	
Sampling					
Consecutive	3	88.62 (82.5–94.74)	0.7462	98 (96.31–99.68)	0.6202
Convenience	4	90.46 (79.63–100)		96.25 (92.53–99.96)	
Initial POCUS performer					
Independent EM residents	5	89.43 (79.31–99.55)	0.7466	96.70 (93.98–99.42)	0.6393
EM residents supervised by attending EP	2	85.72 (77.37–94.06)		97.99 (95.96–100)	

## Discussion

DVT presents a significant healthcare burden; therefore, early diagnosis and the initiation of anticoagulant therapy are essential to reduce the risk of morbidity and mortality as well as prevent complications [35]. Contrast venography is usually considered the “gold standard” for DVT diagnosis; however, point-of-care compression ultrasound is currently regarded as the first-line imaging tool in the emergency department since it is more safe, cost-effective, and non-invasive [36, 37]. The current study shows that both 2 and 3-point POCUS have high sensitivity, specificity, PPV, and NPV for DVT diagnosis. Compared to the 2-point and 3-point ultrasound techniques, the pooled data for other POCUS protocols (Complete compression ultrasound and whole-leg duplex ultrasound) seem to result in higher sensitivity and specificity. Furthermore, our analysis shows that the time from triage to DVT diagnosis when using POCUS in the emergency department is significantly reduced compared to when reference tests are carried out in the radiology department.

The diagnostic results reported in our study are supported by a more recent meta-analysis that compared 2-point and 3-point POCUS and had fewer included studies than ours. The results of that meta-analysis showed high sensitivity and specificity for both 3-point (90% and 95%) and 2-point POCUS (91% and 98%) [38]. Similarly, a previous meta-analysis pooling data for all POCUS protocols (Complete compression ultrasound, 2-point, and 3-point) reported sensitivity and specificity of 95% and 96%, respectively [39]. In addition, a meta-analysis evaluating the accuracy of EP-performed ultrasound reported the ultrasound was able to diagnose DVT with a 94.8% weighted mean sensitivity and 96.2% weighted mean specificity [40]. Despite all these results pointing to high sensitivity and specificity, it should be noted that high heterogeneity exists. Therefore, these findings should be interpreted with caution. We also noticed that some included studies recorded relatively low specificity and sensitivity values. For example, Abbasi and colleagues recorded as low as 41.2% sensitivity for DVT diagnosis [10]. The low accuracy reported in this study was attributed to the fact the POCUS was carried out by second-year emergency residents who had low hours of training. Similarly, Zitek and colleagues recorded a 57.1% sensitivity when using the 2-point protocol [32]. The low sensitivity was also attributed to the fact the operators of that study were less experienced and skilled in ultrasound.

Since training level has been attributed to low diagnostic performance, it is essential to discuss the role of education when carrying out POCUS to diagnose DVT. Our regression analysis showed that the level of training was not a significant source of heterogeneity in the specificity and sensitivity analysis. Moreover, the pooled data shows that POCUS performed by both experienced and inexperienced EP has comparable specificity and sensitivity. However, research shows that inadequate training could result in omission errors, where DVT may not be treated when it is falsely excepted, and commission errors, where anticoagulant therapy is initiated when DVT is falsely confirmed [40]. Even though the exact training and experience required to diagnose DVT is uncertain, The American College of Emergency Physicians guidelines suggests that for clinical decision-making, POCUS training should be done for at least over a two-day course [41]. Furthermore, Blaivas reported that 10 min of training is insufficient for DVT diagnosis but reiterated that when emergency physicians are trained properly, they can accurately diagnose DVT in the emergency department [42]. To support this hypothesis, Blavais and colleagues later reported that 2-h didactic education followed by hands-on training for three hours and previous experience on POCUS has a very high correlation with vascular studies (0.9 kappa and 98% (95% CI: 95.4–100%) agreement). However, the education curriculum currently varies. For this reason, Fox and colleagues called for more uniform and universal training of EP to use POCUS in DVT diagnosis [43].

Our meta-analysis results have also shown that POCUS is advantageous in reducing the time from triage to DVT diagnosis compared to reference tests in the radiology department. These results are reinforced by a Malaysian study of 63 patients, which reported that bedside ultrasound significantly shortened the time between ED arrival and confirmation of DVT (2.24 ± 0.43 h and 17.28 ± 4.77 h, p < 0.001) [44]. In addition, studies claim that POCUS can improve the time to disposition (being discharged from the ED or Hospital). Seyedhosseini and colleagues reported that the time between triage and the disposition of patients was significantly shorter for patients in the emergency department POCUS group compared to the radiologist group (69 min (28–138) vs. 142 min (91–233), respectively; p < 0.001). Similarly, Chu and colleagues reported a significantly shorter disposition time when using bedside ultrasound (p < 0.001) [44]. On the other hand, El-Gazzar and colleagues reported that the time EP took to diagnose DVT was significantly shorter as opposed to the time taken by a radiologist (6.68 ± 1.81 vs. 5.76 ± 1.62 min, respectively; p < 0.001) [20]. Similarly, zitek and colleagues reported that ultrasounds carried out in the ED were completed 84 min before the ultrasound in the radiology department was made available [32]. The significant reduction in time to diagnosis and disposition in ED-performed POCUS reported in these studies can be attributed to the fact that ultrasound devices are usually readily available in the ED for 24 h.

Point-of-care compression ultrasound in DVT diagnosis is also subject to various pitfalls. The first limitation is the location of DVT. Research shows that the 2 and 3-point POCUS protocols cannot diagnose calf vein thrombosis, but whole-leg compression ultrasound carried out in the radiology department can. This means that the 2 or 3-point compression can miss to diagnose some DVTs that would have been detected when using the whole-leg compression technique. However, previous research suggests that the 2-point compression protocol may be as sensitive as the complete compression in diagnosing DVT from the inguinal ligament up to the calf [45]. In addition, DVT in the bedside ultrasound can be mistaken for a Baker’s Cyst or lymph nodes; therefore, it is essential that landmarks such as ensuring the vein is usually closer to the artery are identified. Lymph nodes have also been mistaken for the common femoral vein, thus increasing the rate of false negatives. For instance, Zitek and colleagues reported that a total of 22 false negatives were observed when carrying out the 2-point POCUS, of which one false negative was attributed to the fact that the resident sonographer mistook the lymph node for the common femoral vein, thus contributing to the low sensitivity [32].

In addition, the acute clot has been mistaken for chronic clots. Research shows that an abnormal compression ultrasound may continue to be seen in up to 70% of DVT patients after one year [46]. The thrombus age is usually inferred from the clot echogenicity, of which older clots tend to be more echo dense. However, this skill is generally left to radiologists with advanced skills. Another common error not reported in many studies is inadequate visualization of the popliteal vein. Zitek and colleagues reported that 8 of the 22 false negatives resulted from inadequate popliteal vein visualization [32]. The videos analyzed in that study showed that most residents had mistaken the popliteal vein with the superficial vein. Moreover, one of the residents had mistaken the popliteal vein with a hyperechoic thrombus for the tibial vein. Given this high-frequency error, ultrasound educators should keenly ensure that the learners understand the popliteal vein to help avoid this error in future and improve the diagnostic performance of POCUS.

In the evaluation of DVT, an accurate determination of the pre-test probability for a clot is also crucial. The widely accepted guidelines have recommended using validated scores and D-dimer in evaluating the likelihood of DVT diagnosis among patients bestowing indicative symptoms [47]. As reported in our previous case report of a 51-year-old male with type 2 diabetes and hypertension, after D-dimer testing was done, a clinical judgment suspected DVT as one of the differential diagnoses; thus, doppler ultrasound was carried out and found a distended and non-compressible intramuscular calf muscle which was suggestive of acute thrombosis[48]. Research also shows that using algorithms that incorporate pre-test probability assessment with a sensitive D-dimer test reduces the number of imaging studies carried out [49, 50]. The most commonly validated score system is the Well’s scoring system, of which a ≥ 2 score is indicative of a high pre-test probability of DVT. Studies incorporating a POCUS protocol with Well’s scores and D-dimer testing seem to have a high diagnostic performance. For instance, in the study by Garcia and colleagues, the 3-point ultrasound was led by a well’s criteria and D-dimer testing, and this led to high sensitivity, specificity, and accuracy of 93.2%, 90%, and 91.7%, respectively[9]. Similarly, an Egyptian study conducted the Well’s criteria and D-dimer testing before the 3-point POCUS and found that the sensitivity, specificity, and accuracy of POCUS examination for DVT diagnosis were high (94.12%, 92.42%, and 93.0%, respectively)[20].

Compared to the most recent systematic review and meta-analysis [38] and other two previous meta-analyses [39, 40], our study has more number and most recent studies that assess the role of POCUS in DVT diagnosis. Unlike the study by Lee et al. [38], we were able to evaluate the effect of the level of training on the observed heterogeneity. Our regression analysis showed that the level of training did not contribute to the heterogeneity, and the sensitivity and specificity of both experienced and inexperienced was comparable. This indicates that even EP with the most miniature training can diagnose DVT using POCUS with a certain degree of accuracy. However, the regression analysis also showed that the presence of emergency medicine (EM) attending significantly contributed to heterogeneity, and the pooled specificity was always higher when the EM attending was present. This is to show that even though low training levels can give good outcomes, to obtain better outcomes, it is essential that EM attendings with POCUS experience are present during the POCUS examinations.

### Limitations

The current review was subject to several limitations. First, the eligibility criteria only allowed the inclusion of English-published studies, thus introducing selection bias in our analysis. Secondly, the meta-analysis results showed high heterogeneity; however, the risk of bias assessment revealed a low risk of bias, meaning that the bias did not influence our results. Thirdly, in the meta-regression analysis, we classified the training levels as either experienced or inexperienced and found that the level of training did not influence the heterogeneity. However, the number of hours to train the EP varied from study to study, which, if analyzed, might result in significant heterogeneity. Lastly, very few studies have evaluated the diagnostic performance of the whole-leg compression and complete compression techniques in the emergency department despite our results pointing out that the sensitivity and specificity are higher compared to those of 2-point and 3-point compression techniques. Therefore, further studies should be carried out to support this evidence fully.

## Conclusion

The current meta-analysis has shown that the 2-point, 3-point, complete compression ultrasound, and whole-leg duplex POCUS protocols are excellent in diagnosing DVT in the emergency department. Combining the high diagnostic performance with the fact that POCUS significantly reduces the time from triage to DVT diagnosis, we can recommend that POCUS be utilized as the first-line imaging tool for diagnosing DVT in the emergency department. We also recommend that attending EPs with POCUS experience are present during the DVT diagnosis for better diagnostic performance despite high performance being observed in EPs with less POCUS training.

### Supplementary Information

Supplementary Material 1.

## Data Availability

All data and materials available online or included in the manuscript.
