# Supplementary material for: Deep venous thrombosis (DVT) diagnostics: gleaning insights from point-of-care ultrasound (PoCUS) techniques in emergencies: a systematic review and meta-analysis
Source: Ultrasound J. 2024 Jul 30;16:37. doi: 10.1186/s13089-024-00378-1 (PMC11289207; doi:10.1186/s13089-024-00378-1)
Supplement: Supplementary file 1 — Supplementary Material 1. [file 13089_2024_378_MOESM1_ESM.docx]

**APPENDIX A: Search Strategy**

**PubMed**

[All Fields] ((("Deep vein thrombosis" OR "Deep venous thrombosis" OR "DVT")) AND (("point-of-care ultrasound" OR "bedside ultrasound" OR "POCUS" or "compression sonography" OR "compression ultrasound" OR "2-point ultrasound" OR "3-point ultrasound" OR "whole-leg compression ultrasound" OR "complete compression ultrasound"))) AND (("sensitivity" OR "specificity" OR "Diagnostic value" OR Diagnostic accuracy OR diagnosis OR accuracy).)

[Titles/Abstracts] ((("Deep vein thrombosis" OR "Deep venous thrombosis" OR "DVT")) AND (("point-of-care ultrasound" OR "bedside ultrasound" OR "POCUS" or "compression sonography" OR "compression ultrasound" OR "2-point ultrasound" OR "3-point ultrasound" OR "whole-leg compression ultrasound" OR "complete compression ultrasound"))) AND (("sensitivity" OR "specificity" OR "Diagnostic value" OR Diagnostic accuracy OR diagnosis OR accuracy).)

**Google Scholar**

[with all of the words] (Deep vein thrombosis OR Deep venous thrombosis OR venous thrombosis OR venous thromboembolism OR DVT)

[With at least one of the words] (point-of-care ultrasound OR bedside ultrasound OR POCUS or compression sonography OR compression ultrasound OR 2-point ultrasound OR 3-point ultrasound OR whole-leg compression ultrasound OR complete compression ultrasound) AND (sensitivity OR specificity OR Diagnostic value OR diagnostic accuracy OR accuracy OR Diagnosis)

**ScienceDirect**

[Title, abstract or author-specified keywords] (Deep vein thrombosis OR Deep venous thrombosis OR venous thrombosis OR DVT) AND (point-of-care ultrasound OR bedside ultrasound OR POCUS OR 2-point ultrasound OR 3-point ultrasound)

[Title] (Deep vein thrombosis OR Deep venous thrombosis OR venous thrombosis OR DVT) AND (point-of-care ultrasound OR bedside ultrasound OR POCUS)

**Embase**

(“Deep vein thrombosis” OR “Deep venous thrombosis” OR “DVT”) AND (“point-of-care ultrasound” OR “bedside ultrasound” OR “POCUS” or “compression sonography” OR “compression ultrasound” OR “2-point ultrasound” OR “3-point ultrasound” OR “whole-leg compression ultrasound” OR “complete compression ultrasound”) AND (“sensitivity” OR “specificity” OR “Diagnostic value”).
